# Low-Noise Direct Denoising Sampling for DPM++ Samplers with Denoised Outputs

**DOI:** 10.3390/s26206480

**Published:** 2026-10-13

**Authors:** Zhigang Liang, Jian Wang

**Affiliations:** The College of Computer Science and Technology, Jilin University, No. 2699 Qianjin Street, Changchun 130012, China; liangzg22@mails.jlu.edu.cn

**Keywords:** diffusion models, DPM++ samplers, denoised output, low-noise sampling, image generation, traffic scenario generation, autonomous driving

## Abstract

Diffusion models have been widely used in image generation and structured trajectory generation. DPM++ samplers improve few-step inference by recursively propagating current and historical denoised states, but their behavior in the low-noise regime remains less examined. This paper studies whether the recursive low-noise update is always necessary when the model-predicted denoised output has become relatively stable. Based on this analysis, we propose Low-Noise Direct Denoising Sampling (LNDDS), a training-free modification that replaces the recursive DPM++ update with the current denoised prediction during selected low-noise steps. LNDDS keeps the model architecture, training objective, noise schedule, number of sampling steps, and number of function evaluations unchanged. Experiments on MJHQ-30K image generation and HiLo–nuPlan traffic scenario generation show that the effect of LNDDS is domain-dependent. LNDDS does not provide consistent quality improvements for image generation under the evaluated setting, but it shows clear benefits for traffic scenario generation by improving trajectory quality, motion smoothness, and physical validity. Mechanism analysis further shows that low-noise direct updates affect sampling trajectories differently across domains. These results suggest that LNDDS is a task-dependent low-noise state-update strategy that is more suitable for structured trajectory generation than for general text-to-image generation.

## 1. Introduction

Diffusion models have recently become an important paradigm in generative modeling and have demonstrated strong modeling capabilities in tasks such as image generation, video generation, trajectory prediction, and traffic scenario generation. The fundamental idea of diffusion models is to gradually perturb real data through a forward diffusion process and learn a progressive denoising mapping from the noise distribution to the data distribution during the reverse process. Existing studies have systematically formulated diffusion models from the perspectives of probabilistic modeling, continuous-time stochastic processes, and score matching, enabling diffusion models to evolve into a generative framework with stable training procedures and strong distribution modeling capabilities [1,2,3]. With the advancement of model architectures and training strategies, the application scope of diffusion models has expanded from early image generation tasks to high-resolution visual generation, video generation, multi-agent trajectory prediction, and autonomous driving traffic scenario generation [4,5,6,7,8]. For these tasks, generation quality depends not only on the modeling capability of the trained network but also strongly on the numerical behavior of the reverse sampling process [9,10,11]. In particular, for structured traffic scenario generation, errors accumulated during reverse sampling can directly affect trajectory continuity, multi-agent interaction rationality, and physical consistency [6,7].

Diffusion models typically require multiple iterative denoising steps to complete generation, making inference efficiency a long-standing limitation for practical deployment. To reduce sampling steps while maintaining generation quality, previous studies have proposed efficient samplers from perspectives such as non-Markovian sampling, pseudo numerical methods, high-order ODE solvers, and unified predictor-corrector frameworks [10,11,12,13,14]. These methods generally formulate the reverse diffusion process as a numerical solution problem of a continuous dynamical system or diffusion ODE, and improve few-step sampling performance through multi-step recursion, high-order approximation, or correction mechanisms. Among them, DPM-Solver++ (DPM++) [11] has become a widely used efficient sampler in practical diffusion-generation systems. It formulates reverse diffusion as the numerical integration of a diffusion ODE using model-predicted denoised outputs, while its multi-step variants reuse historical denoised predictions to improve stability and accuracy under few-step sampling settings. However, existing sampling methods mainly focus on numerical accuracy along the overall sampling trajectory, sampling step reduction, and global error control, while the internal update behavior of samplers at different noise stages remains insufficiently analyzed.

This paper focuses on the sampling behavior of DPM++ samplers in the low-noise regime. Existing samplers generally employ a unified recursive update strategy throughout the entire noise trajectory. However, in the low-noise stage, the current sample has already approached the data manifold, and the model-predicted denoised output gradually becomes stable. At this point, continuing to rely on historical sampling states from previous steps for recursive updates may introduce redundant dependence on residual noisy states. Since historical states are not completely clean, the remaining noise errors may continue to propagate during the later inference stages and affect the final generation results. In image generation tasks, such low-noise state changes may affect local appearance and fine-scale details, although such changes do not necessarily improve overall image quality. In traffic scenario generation tasks, they may manifest as trajectory oscillations, discontinuous motion patterns, unstable interactions, and reduced physical consistency. Based on this observation, this paper investigates the update mechanism in the low-noise stage, analyzes the relationship between model-predicted denoised outputs and DPM++ historical recursive terms, and proposes a Low-Noise Direct Denoising Sampling (LNDDS) method. When the noise level falls below a predefined threshold, the proposed method skips the recursive update of DPM++ and directly uses the model-predicted denoised output as the next sampling state, thereby reducing the propagation of residual noise through historical states in the low-noise regime.

## 2. Related Works

### 2.1. Diffusion Models and Applications

Diffusion models have become an important approach to generative modeling. DDPM establishes a probabilistic generation framework based on a forward noising process and a learned reverse denoising process [1]. Score-based Generative Modeling further provides a continuous-time formulation that unifies diffusion models through stochastic differential equations and probability flow ODEs [3]. These foundations have enabled diffusion models to be applied to multiple generation tasks, including high-resolution image synthesis [4], video generation [15], and structured multi-agent motion and traffic scenario generation [6,7].

In image generation, Latent Diffusion Models improve computational efficiency by performing the diffusion process in a learned latent space [4], while DiT improves model scalability by replacing the conventional U-Net backbone with a Transformer architecture [5]. Video Diffusion Models extend image-based diffusion architectures to jointly model spatial appearance and temporal dynamics, enabling the generation of temporally coherent video sequences [15]. For image generation, the generated object can generally be represented as $x_{0}^{\mathrm{img}}\in R^{H\times W\times C}$, and the model learns the conditional distribution $p_{\theta }\left(x_{0}^{\mathrm{img}}|c\right)$, where *c* denotes a text prompt, class label, or other conditioning information.

Beyond visual-content generation, diffusion models have increasingly been applied to structured spatiotemporal tasks, particularly multi-agent trajectory prediction and autonomous-driving traffic scenario generation. MotionDiffuser models the joint distribution of multiple agents’ future trajectories and supports controllable trajectory sampling through differentiable constraints [6]. Scenario Diffusion combines latent diffusion with map and scenario conditions to generate controllable agent poses, orientations, and trajectories for autonomous-driving simulation [7]. In these tasks, a generated traffic scenario can be represented as $Y={\left\{s_{i}^{t}\right\}}_{i=1:N,\;t=1:T_{f}}$, where $s_{i}^{t}=\left(x_{i}^{t},y_{i}^{t},\theta_{i}^{t},v_{i}^{t},\ldots \right)$ denotes the motion state of the *i*-th agent at the *t*-th future timestep. Given historical trajectories *H* and map information *M*, the diffusion model learns the conditional distribution $p_{\theta }\left(Y|H,M\right)$ to generate diverse future motions, controllable driving scenarios, and realistic multi-agent interactions [6,7].

Compared with image generation, traffic scenario generation imposes additional requirements on temporal continuity, agent-interaction rationality, map compliance, and physical consistency [6,7]. Across these application domains, generation quality depends not only on the network architecture and training objective but also on the numerical behavior of the reverse sampling process [10,11]. In contrast to existing task-oriented studies, this paper focuses on the inference-time behavior of DPM++(2M) in the low-noise regime and investigates how its recursive state-update rule affects the final generation quality.

### 2.2. Efficient Diffusion Samplers

Following this ODE perspective, DPM-Solver and DPM-Solver++ formulate diffusion sampling as a numerical solution problem of the diffusion ODE and improve few-step generation through high-order solvers [10,11]. DPM-Solver++ is designed for guided sampling and operates on model-predicted denoised outputs. It provides first-order, higher-order single-step, and higher-order multi-step variants, including DPM++(2S), DPM++(2M), and DPM++(3M). The single-step variants use intermediate model evaluations within each solver step, whereas the multi-step variants reuse denoised predictions from previous steps. This work focuses on the deterministic DPM++(2M) sampler, which is widely used for guided text-to-image generation and is adopted by Playground v2.5. UniPC further provides a unified predictor–corrector framework that improves the order of accuracy without additional model evaluations [14]. These methods have significantly improved diffusion inference efficiency and have become important components of modern diffusion-generation pipelines.

Despite their effectiveness, most existing efficient samplers mainly focus on global numerical accuracy, sampling step reduction, and quality-efficiency trade-offs along the entire denoising trajectory. The internal behavior of recursive samplers at different noise levels remains less explored. In particular, DPM++ samplers continue to propagate historical denoising states in the low-noise regime, where the model-predicted denoised output tends to become increasingly stable and close to the final clean sample. This motivates us to investigate whether such low-noise recursive updates introduce redundant dependence on residual noisy states.

## 3. Low-Noise Direct Denoising Sampling

This section presents the proposed Low-Noise Direct Denoising Sampling (LNDDS) strategy for DPM++ diffusion inference. We first revisit the denoised-output formulation and the recursive state update of DPM++ samplers. We then analyze the behavior of DPM++ updates in the low-noise regime and show that the recursive state carry may introduce redundant dependence on residual noisy states. Based on this analysis, we propose to replace the low-noise DPM++ update with the model-predicted denoised output when the noise level becomes sufficiently small.

### 3.1. Problem Formulation

Let $x_{i}\in R^{d}$ denote the sampling stateat noise level $\sigma_{i}$, where *d* is the state dimension. We use *N* sampling transitions, indexed by i=0,…,N−1, to transform $x_{0}$ into the final output $x_{N}$. The model is evaluated at the decreasing positive noise levels

(1) $$\sigma_{0}>\sigma_{1}>\cdots >\sigma_{N-1}>0,$$

The last transition produces $x_{N}$; when an implementation appends a terminal zero noise level, it is understood as the standard zero-noise-limiting update. This convention avoids evaluating logarithms at zero in the interior DPM-Solver++(2M) formulas below. Given a trained diffusion model, inference gradually transforms the initial noisy sample $x_{0}$ into an estimate of the clean sample through these *N* updates. We define

(2) $$D_{\theta }:R^{d}\times R_{+}\times C\to R^{d},$$

as the model-predicted denoising function, where θ denotes the fixed parameters of the trained diffusion model and C denotes the space of optional conditioning information. Given a noisy sample $x_{i}$, its noise level $\sigma_{i}$, and conditioning information *c*, the function $D_{\theta }\left(x_{i},\sigma_{i};c\right)$ predicts the corresponding clean sample $x_{\mathrm{clean}}$. Thus, the three arguments of Equation (2) are the current state, its noise level, and the condition, while the output has the same dimension as the state. For notational simplicity, the fixed conditioning variable *c* is omitted in the following derivations.

At sampling step *i*, we denote the model-predicted denoised output by

(3) $$D_{i}:=D_{\theta }\left(x_{i},\sigma_{i}\right),$$

Thus, $D_{i}\in R^{d}$ is the model’s current estimate of the clean sample at noise level $\sigma_{i}$, rather than the updated sampling state $x_{i+1}$. In this paper, we focus on samplers, such as DPM++, that explicitly use this model-predicted denoised output in their state-update rules.

We focus on the low-noise regime, where $\sigma_{i}$ is close to zero. At this stage, the current sample has already approached the data manifold, and the model-predicted denoised output tends to become increasingly stable. However, DPM++ still performs recursive updates based on the current sampling state and historical denoising states. Since these states may contain residual errors, such recursive updates may continue to propagate state deviations into the final denoising stage.

### 3.2. Denoised Outputs and DPM++ State Carry

DPM++ samplers update the sampling state through a recursive numerical solver. For an interior deterministic DPM-Solver++(2M) step with i≥1 and $\sigma_{i+1}>0$, the next state can be written in the denoised-output form

(4) $$x_{i+1}=\alpha_{i}x_{i}+\left(1-\alpha_{i}\right){\tilde{D}}_{i},\;\alpha_{i}=\frac{\sigma_{i+1}}{\sigma_{i}},$$

where $\alpha_{i}\in \left(0,1\right)$ is the fraction of the current noisy state carried to the next step and $1-\alpha_{i}$ is the weight assigned to the extrapolated denoised estimate ${\tilde{D}}_{i}$. For the second-order multi-step update, this estimate is

(5) $$\begin{array}{cc} {\tilde{D}}_{i}\; & =\left(1+\frac{1}{2r_{i}}\right)D_{i}-\frac{1}{2r_{i}}D_{i-1},\\ r_{i}\; & =\frac{h_{i-1}}{h_{i}},\\ h_{i}\; & =\log\frac{\sigma_{i}}{\sigma_{i+1}}, \end{array}$$

Here, $D_{i}=D_{\theta }\left(x_{i},\sigma_{i}\right)$ denotes the current model-predicted denoised output, and $D_{i-1}$ denotes the denoised output from the previous sampling step. The positive log-noise interval $h_{i}$ is the step size from $\sigma_{i}$ to $\sigma_{i+1}$, and $r_{i}$ is the ratio of the preceding interval to the current interval. Consequently, the two coefficients in Equation (5) specify how the current and previous denoised predictions are combined. At the first transition, where $D_{i-1}$ is unavailable, the standard first-order initialization is used; a terminal zero-noise transition is handled by the solver’s standard limiting rule. Our analysis concerns the later low-noise multi-step updates for which Equation (5) is defined.

Equations (4) and (5) show that DPM++ does not only depend on the current denoised output, but also explicitly uses the current recursive state $x_{i}$ and historical denoising information $D_{i-1}$. This stateful property is beneficial for high-order numerical accuracy, especially under few-step sampling settings. However, in the low-noise regime, when the denoised prediction has become relatively stable, continuing to propagate historical states may introduce redundant dependence on residual noisy states.

### 3.3. Low-Noise Error Propagation

We analyze how state deviations and denoising errors propagate through the DPM-Solver++(2M) recursion in the low-noise regime. Let $\sigma_{\tau }>0$ denote a predefined low-noise threshold. The first index inside this regime is

(6) $$i^{★}=\min\left\{i:\sigma_{i}\leq \sigma_{\tau }\right\},$$

Thus, all updates with $i\geq i^{★}$ use noise levels at or below the selected threshold. The state entering the low-noise regime is decomposed as

(7) $$x_{i^{★}}=x_{i^{★}}^{*}+\delta_{i^{★}},$$

where $x_{i^{★}}^{*}$ denotes a reference low-noise state and $\delta_{i^{★}}$ denotes the residual state deviation accumulated before the low-noise boundary. This residual deviation may contain discretization errors, denoising prediction errors, and perturbations propagated from previous sampling steps.

To quantify its subsequent propagation, let $x_{i}^{*}$ and $D_{i}^{*}$ denote a reference state and its associated reference denoised output, respectively. We define the state and denoised-output deviations as

(8) $$e_{i}=x_{i}-x_{i}^{*},\;\varepsilon_{i}=D_{i}-D_{i}^{*},$$

In particular, $e_{i^{★}}=\delta_{i^{★}}$. For the DPM++ recursion analyzed next, the reference trajectory follows the same noise schedule and DPM-Solver++(2M) coefficients as the actual trajectory. Hence, $e_{i}$ measures a perturbation between two trajectories governed by the same update rule, rather than the unknown error with respect to a ground-truth sample.

Subtracting the reference update from the actual update in Equations (4) and (5) gives the state-error recursion

(9) $$\begin{array}{cc} e_{i+1}= & \alpha_{i}e_{i}+\left(1-\alpha_{i}\right)\left[\left(1+\frac{1}{2r_{i}}\right)\varepsilon_{i}-\frac{1}{2r_{i}}\varepsilon_{i-1}\right], \end{array}$$

Equation (9) identifies two error-propagation channels. First, the state-carry term $\alpha_{i}e_{i}$ recursively transfers the current state deviation. Second, the multi-step term involving $\varepsilon_{i-1}$ transfers the deviation of the previous denoised prediction across sampling steps.The term involving $\varepsilon_{i}$ is the effect of the current denoised-output deviation. Equation (9) is an identity under the paired-trajectory construction above; the inequalities below arise only after norm bounds are applied.

We assume that the denoising map is locally Lipschitz around the reference trajectory:

(10) $$∥\varepsilon_{i}{∥}_{2}\;\leq \;L_{i}{∥e_{i}∥}_{2}+b_{i},$$

where $L_{i}$ measures the local sensitivity of the denoising map to its input state, and $b_{i}\geq 0$ accounts for any discrepancy between the model prediction at the reference state and the selected reference denoised output. If $D_{i}^{*}=D_{\theta }\left(x_{i}^{*},\sigma_{i}\right)$, local Lipschitz continuity gives the bound with $b_{i}=0$. Otherwise, $b_{i}$ absorbs that reference or model-approximation mismatch. Separating $L_{i}$ and $b_{i}$ distinguishes amplification of an input-state perturbation from an additive discrepancy.

Applying the triangle inequality to Equation (9) gives

(11) $$∥e_{i+1}{∥}_{2}\;\leq \;A_{i}∥e_{i}{∥}_{2}\;+\;B_{i}{∥e_{i-1}∥}_{2}+c_{i},$$

where

(12) $$\begin{array}{cc} A_{i}= & |\alpha_{i}|+|1-\alpha_{i}|\left|1+\frac{1}{2r_{i}}\right|L_{i},\\ B_{i}= & |1-\alpha_{i}|\left|\frac{1}{2r_{i}}\right|L_{i-1},\\ c_{i}= & |1-\alpha_{i}|\left[\left|1+\frac{1}{2r_{i}}\right|b_{i}+\left|\frac{1}{2r_{i}}\right|b_{i-1}\right], \end{array}$$

All three coefficients are nonnegative. Here, $A_{i}$ quantifies propagation through the state-carry and current denoising terms; $B_{i}$ quantifies the additional dependence on the previous step; and $c_{i}$ collects the additive discrepancies $b_{i}$ and $b_{i-1}$. Therefore, a non-negligible $B_{i}$ can continue to carry historical deviations into the final sampling steps.

For a multi-step interpretation, define

(13) $$u_{i}=\left[\begin{array}{c} ∥e_{i}{∥}_{2}\\ ∥e_{i-1}{∥}_{2} \end{array}\right],\;M_{i}=\left[\begin{array}{cc} A_{i} & B_{i}\\ 1 & 0 \end{array}\right],\;q_{i}=\left[\begin{array}{c} c_{i}\\ 0 \end{array}\right],$$

Equation (11) can then be expressed component-wise as

(14) $$u_{i+1}\leq M_{i}u_{i}+q_{i},$$

where ≤ denotes element-wise inequality. The augmented vector $u_{i}$ stores the current and previous error magnitudes, $M_{i}$ advances this two-step recursion, and $q_{i}$ injects the additive discrepancy. After *m* low-noise DPM++ updates, repeated application yields

(15) $$\begin{array}{cc} u_{i^{★}+m}\leq & M_{i^{★}+m-1}\cdots M_{i^{★}}u_{i^{★}}\\ \; & +\sum_{j=0}^{m-1}\left(M_{i^{★}+m-1}\cdots M_{i^{★}+j+1}\right)q_{i^{★}+j}, \end{array}$$

where an empty matrix product is interpreted as the identity matrix. Equation (15) quantifies how the initial residual deviation $\delta_{i^{★}}$ and subsequent denoising errors propagate through the remaining low-noise steps. In particular, the product of the matrices $M_{i}$ determines whether the initial deviation is attenuated or amplified, whereas each summand propagates the discrepancy introduced at step $i^{★}+j$ through all later steps. Matrix products are ordered chronologically from right to left.

In contrast, LNDDS directly sets the next state to the current denoised prediction. To distinguish this trajectory from the DPM++ trajectory above, we use the superscript *L*:

(16) $$x_{i+1}^{L}=D_{\theta }\left(x_{i}^{L},\sigma_{i}\right),$$

Let $x_{i+1}^{*,L}=D_{i}^{*}$, $e_{i}^{L}=x_{i}^{L}-x_{i}^{*,L}$, and $\varepsilon_{i}^{L}=D_{\theta }\left(x_{i}^{L},\sigma_{i}\right)-D_{i}^{*}$. Relative to this rule-matched reference, the local error satisfies

(17) $${∥e_{i+1}^{L}∥}_{2}\;=\;∥\varepsilon_{i}^{L}{∥}_{2}\;\leq \;L_{i}{∥e_{i}^{L}∥}_{2}+b_{i},$$

Compared with Equation (11), the LNDDS update removes both the explicit state-carry coefficient $\alpha_{i}$ and the historical error term associated with $e_{i-1}$. It therefore prevents previous denoised outputs from being explicitly reused after the low-noise switching point. Because the bounds use reference trajectories matched to their respective update rules, this comparison isolates propagation structure; it does not by itself prove lower error with respect to the data distribution.

We next analyze how the number of direct-denoising steps affects the propagation of low-noise errors. Recall that *N* is the total number of sampling transitions, and let K∈{0,1,…,N} denote the number of final transitions using LNDDS. The baseline is K=0. For K≥1, the switching index is

(18) $$i_{\tau }=N-K,$$

Therefore, selecting K≥1 under a fixed schedule is equivalent to selecting the low-noise threshold

(19) $$\sigma_{\tau }=\sigma_{N-K},$$

For comparisons across different total numbers of sampling steps, we additionally define the normalized direct-denoising ratio as

(20) $$\rho =\frac{K}{N},$$

Here, ρ∈[0,1] is the fraction of sampling transitions replaced by direct denoising. Reporting both *K* and ρ avoids interpreting the same absolute *K* as the same range under different discretizations.

Repeatedly applying Equation (17) over the final *K* sampling steps gives

(21) $$\begin{array}{cc} {∥e_{N}^{L}∥}_{2}\leq & \left(\prod_{i=N-K}^{N-1}L_{i}\right){∥e_{N-K}^{L}∥}_{2}\\ \; & +\sum_{j=N-K}^{N-1}\left(\prod_{\ell =j+1}^{N-1}L_{\ell }\right)b_{j}, \end{array}$$

where an empty product is interpreted as one. The first term in Equation (21) describes the propagation of the LNDDS-referencedeviation present at the switching point, while the second term describes the accumulation of denoising approximation errors over the selected *K* steps. Each $b_{j}$ is multiplied by the sensitivities of all subsequent direct-denoising steps.

For a more interpretable special case, suppose that

(22) $$L_{i}\leq \bar{L},\;b_{i}\leq \bar{b},\;i=N-K,\ldots ,N-1,$$

If $0\leq \bar{L}<1$, then

(23) $${∥e_{N}^{L}∥}_{2}\leq {\bar{L}}^{K}{∥e_{N-K}^{L}∥}_{2}+\bar{b}\frac{1-{\bar{L}}^{K}}{1-\bar{L}},$$

Equation (23) shows that increasing *K* can attenuate the state deviation at the switching boundary when the denoising map is locally contractive. However, increasing *K* also includes earlier and potentially less stable denoising steps and accumulates more model-approximation errors. If some of the selected steps have $L_{i}\geq 1$, or if their corresponding $b_{i}$ values are large, the contraction condition no longer holds and the final error may increase. The boundary term decays as ${\bar{L}}^{K}$, whereas the additive term approaches the error floor $\bar{b}/\left(1-\bar{L}\right)$. Thus, even in the contractive case, increasing *K* helps only when boundary-error reduction outweighs the additional accumulated discrepancy.

Consequently, the error bound is not necessarily monotonic with respect to *K*. A small *K* may not sufficiently remove the historical-state dependence of DPM-Solver++(2M), whereas an excessively large *K* may switch to direct denoising before the denoised prediction has become stable. The appropriate value of *K* therefore depends on the local stability of the denoising model, the noise schedule, the total number of sampling steps, and the structure of the generation task.

The comparison between Equation (11) and (21) do not imply that LNDDS always has a smaller global sampling error than DPM-Solver++(2M). Direct denoising is expected to be beneficial only when the selected low-noise denoising maps are sufficiently stable and the historical-error contribution $B_{i}{∥e_{i-1}∥}_{2}$ is larger than the approximation error introduced by the direct updates. If multi-step corrections are required to recover fine-scale information, increasing *K* may instead degrade sample quality. This conditional result is consistent with the different behaviors observed in image generation and structured traffic trajectory generation.

The domain difference may also be interpreted from a frequency perspective, although the present experiments do not establish a causal frequency-domain mechanism. In images, low-noise corrections may continue to refine spatial high-frequency content such as edges and textures; removing them too early can reduce fidelity. In traffic trajectories, some temporal high-frequency variation instead corresponds to acceleration oscillation or jitter, so suppressing it can improve motion regularity. This interpretation further motivates selecting *K* separately for each domain.

Because the reference state $x_{i}^{*}$, the local sensitivity $L_{i}$, and the approximation error $b_{i}$ are unavailable during practical sampling, we use observable quantities as empirical proxies. The denoised-prediction change is defined as

(24) $$C_{i}=\frac{∥D_{i}-D_{i-1}{∥}_{2}}{∥D_{i}{∥}_{2}+\epsilon },$$

and the DPM++ update deviation is defined as

(25) $$\Delta_{i}=\frac{{∥x_{i+1}^{\mathrm{DPM}++}-D_{i}∥}_{2}}{∥D_{i}{∥}_{2}+\epsilon },$$

Here, ϵ>0 is a fixed constant that prevents division by zero. The quantity $C_{i}$ measures the relative change between consecutive denoised predictions, whereas $\Delta_{i}$ measures the relative correction introduced by the DPM++ update, with $x_{i+1}^{\mathrm{DPM}++}$ computed by Equations (4)–(5). Both are dimensionless diagnostics; neither is a sample-quality metric or a guarantee that switching will improve the output.

In practice, we recommend selecting *K* on a held-out validation set. Given *M* validation samples, the average denoised-prediction change at step *i* is

(26) $${\bar{C}}_{i}=\frac{1}{M}\sum_{m=1}^{M}C_{i}^{\left(m\right)},$$

where $C_{i}^{\left(m\right)}$ is computed for validation sample *m*. Averaging yields a step-wise stability profile for the target domain. A candidate low-noise switching point can then be identified as

(27) $$i_{\tau }^{\mathrm{cand}}=\min\left\{i:{\bar{C}}_{j}\leq \tau_{C},\;\forall j=i,\ldots ,N-1\right\},$$

where $\tau_{C}>0$ is determined using validation data. The universal quantifier requires stability at every subsequent step. If the set in Equation (27) is empty, we set $K_{\mathrm{cand}}=0$ and retain the baseline. Otherwise, the maximum candidate range is

(28) $$K_{\mathrm{cand}}=N-i_{\tau }^{\mathrm{cand}},$$

Thus, $K_{\mathrm{cand}}$ is a screening bound, not a claim that the largest admissible *K* is optimal. The final value is selected from the candidate set using a domain-specific validation objective:

(29) $$K^{*}=\underset{K\in K_{\mathrm{cand}}}{\arg\min}J_{\mathrm{val}}\left(K\right),$$

where $K_{\mathrm{cand}}=\left\{K\in K:K\leq K_{\mathrm{cand}}\right\}$, and K is a prespecified finite set including K=0. The scalar $J_{\mathrm{val}}$ is the primary domain-specific validation objective; if metrics are aggregated, their weights must be fixed before test-set evaluation. Once selected, $K^{*}$ is held fixed for testing.

This procedure should be interpreted as a practical selection guideline rather than a universally optimal rule. Image generation and structured trajectory generation may enter stable denoising regimes at different noise levels and may assign different importance to low-noise corrections. Therefore, the effective direct-denoising range must be determined separately for each domain. The step-wise and aggregated behaviors of $C_{i}$, $\Delta_{i}$, and different values of *K* are evaluated in Section 4.

### 3.4. Low-Noise Direct Denoising Sampling

Based on the above analysis, we propose Low-Noise Direct Denoising Sampling (LNDDS). The core idea is simple: when the noise level is higher than a predefined threshold, we keep the standard DPM++ update; when the noise level falls below the threshold, we skip the recursive DPM++ update and directly use the model-predicted denoised output as the next sampling state. Formally, the proposed sampling rule is

(30) $$x_{i+1}=\left\{\begin{array}{cc} S_{\mathrm{DPM}++}\left(x_{i},\sigma_{i},\sigma_{i+1};D_{i-1}\right),\; & \sigma_{i}>\sigma_{\tau },\\ D_{\theta }\left(x_{i},\sigma_{i}\right),\; & \sigma_{i}\leq \sigma_{\tau }, \end{array}\right.$$

where $S_{\mathrm{DPM}++}$ denotes the standard DPM++ update and $\sigma_{\tau }$ is the switching threshold. The semicolon separates the historical output $D_{i-1}$ from the current state and noise levels; the current output $D_{i}$ is given by Equation (3). Equivalently, for the last *K* transitions, the first LNDDS update is at i=N−K.

The proposed strategy removes the historical-state dependency of DPM++ in the low-noise regime. In Equation (30), the low-noise update becomes a local denoising step that only depends on the current sample and noise level (and the fixed condition omitted from the notation). Therefore, it avoids the explicit recursive state-carry term $\alpha_{i}x_{i}$ and the multi-step extrapolation term involving $D_{i-1}$. This reduces the repeated propagation of residual state deviations through recursive low-noise updates.

It is important to note that LNDDS is not an early stopping strategy. The sampling process still proceeds through the same noise schedule and uses the same number of denoising steps. The only difference is the state-update rule used after the noise level falls below $\sigma_{\tau }$. Therefore, LNDDS does not require any modification to the network architecture, training objective, or learned parameters. It only changes the sampling rule during inference and is compatible with diffusion samplers that provide a model-predicted denoised output. In image generation, this modification may change local appearance and fine-scale details, although such changes do not necessarily translate into better distribution-level quality. In traffic scenario generation, it can help reduce trajectory jitter and improve motion continuity under the evaluated setting.

## 4. Experiments

We evaluate LNDDS in two domains: text-to-image generation and multi-agent traffic scenario generation. The goal is not to change the model architecture or training objective, but to test the effect of replacing the low-noise DPM++ recursive state update with the current model-predicted denoised output during inference. The baseline and LNDDS use the same model weights, conditions, random seeds, initial noise, sigma schedule, number of sampling steps, and number of network evaluations. Therefore, the reported differences are caused only by the low-noise state-update rule.

In the following tables, LNDDS-*K* denotes using direct denoising in the last *K* low-noise sampling steps, while the baseline, equivalently LNDDS-0, keeps the original DPM++ update for all sampling steps. We evaluate K∈{2,5,7,10}. Under a fixed sigma schedule, selecting the last *K* sampling steps is equivalent to choosing $\sigma_{\tau }$ as the largest noise level among those steps. Because the total number of sampling steps can vary, we interpret *K* together with the normalized switching ratio K/N, where *N* is the total number of inference steps.

### 4.1. Experimental Setup

#### 4.1.1. Image Generation

We use Playground v2.5 [16] as the text-to-image generation model. The model weights, text encoders, variational autoencoder, classifier-free guidance configuration, scheduler configuration, and sigma schedule are kept unchanged for all methods. The evaluation is conducted on MJHQ-30K [16], which contains 30,000 prompts and corresponding reference images across ten semantic categories. For each prompt, all methods use the same random seed and the same initial latent. The default image resolution is 1024×1024, the guidance scale is 3.0, and the number of inference steps is 30.

#### 4.1.2. Traffic Scenario Generation

For traffic scenario generation, we use HiLo, a diffusion-based multi-agent scenario generation model trained on approximately one million nuPlan [17] training samples. A single HiLo checkpoint is used for all experiments, and LNDDS is applied only during inference. The evaluation is performed on a held-out nuPlan test set containing 15,000 scenarios. For each scenario, all methods use the same historical trajectories, map inputs, agent masks, random seed, initial noise, sigma schedule, and number of denoising steps. Unless otherwise stated, the main traffic results use 20 inference steps.

The experimental protocols for both image generation and traffic scenario generation, including the models, datasets, evaluation sizes, inference steps, and compared methods, are summarized in Table 1.

### 4.2. Evaluation Metrics

#### 4.2.1. Image Metrics

We report five image generation metrics. FID [18] and CMMD [19] evaluate distributional consistency between generated and reference images, where lower values are better. ImageReward [20] (IR) estimates prompt-conditioned human preference, where higher values are better. CLIP Score [21] (CS) measures text–image semantic alignment, and Aesthetic Score [22] (AS) estimates visual appeal; higher values are better for both metrics.

#### 4.2.2. Traffic Metrics

For trajectory accuracy, we report average displacement error (ADE) and final displacement error (FDE):

(31) $$\begin{array}{cc} \mathrm{ADE} & =\frac{1}{AT}\sum_{a=1}^{A}\sum_{t=1}^{T}{∥{\hat{p}}_{a}^{t}-p_{a}^{t}∥}_{2}, \end{array}$$

(32) $$\begin{array}{cc} \mathrm{FDE} & =\frac{1}{A}\sum_{a=1}^{A}{∥{\hat{p}}_{a}^{T}-p_{a}^{T}∥}_{2}, \end{array}$$

Here, *A* is the number of valid agents, *T* is the number of predicted time steps, and ${\hat{p}}_{a}^{t}$ and $p_{a}^{t}$ are the generated and recorded two-dimensional positions of agent *a* at time *t*. Equation (31) averages over all valid agent–time pairs, whereas Equation (32) evaluates only the last predicted time step. We use *A*, rather than *N*, to avoid conflict with the number of sampling transitions in Section 3. Wealso report mean jerk, a smoothness score, and vehicle collision rate. Lower values are better for all traffic metrics in the reported tables.

#### 4.2.3. Low-Noise Mechanism Metrics

To inspect low-noise sampling, we record three dimensionless diagnostics of the intermediate states. The denoised-prediction change from Equation (24) is evaluated as

(33) $$C_{i}=\frac{{∥D_{i}-D_{i-1}∥}_{2}}{{∥D_{i}∥}_{2}+\epsilon },$$

where a smaller value indicates that consecutive model-predicted denoised outputs are closer. The DPM++ update deviation from Equation (25) is evaluated as

(34) $$\Delta_{i}=\frac{{∥x_{i+1}^{\mathrm{DPM}++}-D_{i}∥}_{2}}{{∥D_{i}∥}_{2}+\epsilon },$$

which measures the relative distance between the original DPM++ next state and the direct-denoising state $D_{i}$. Finally, the cross-sampler state divergence is

(35) $$E_{i}=\frac{{∥x_{i}^{L}-x_{i}^{B}∥}_{2}}{{∥x_{i}^{B}∥}_{2}+\epsilon },$$

where $x_{i}^{B}$ and $x_{i}^{L}$ are paired baseline and LNDDS states obtained using the same condition and initial noise. The constant ϵ>0 is the same as in Equations (24) and (25). The metric $E_{i}$ measures the accumulated relative state difference and is zero before the samplers diverge. None of $C_{i}$, $\Delta_{i}$, and $E_{i}$ directly measures perceptual quality, trajectory accuracy, or physical validity.

### 4.3. Main Results

#### 4.3.1. Image Generation on MJHQ-30K

Table 2 shows that the baseline obtains the best FID, CMMD, and IR on MJHQ-30K. With K=2, CS and AS increase from 29.348 and 6.817 to 29.472 and 6.861, respectively, while FID, CMMD, and IR become worse. The LNDDS-5 setting obtains the best CS (29.648) and AS (6.921), but further degrades the three distribution- and preference-level metrics. Increasing *K* to 7 and 10 produces progressively larger degradation, with FID increasing to 19.107 and 30.390, respectively. These results reveal a non-monotonic, metric-dependent trend: a small or moderate direct-denoising range can slightly improve semantic alignment and aesthetics, whereas an excessively large *K* substantially degrades the overall image distribution.

#### 4.3.2. Traffic Scenario Generation on nuPlan

Table 3 summarizes the traffic generation results. As *K* increases from 2 to 5, 7, and 10, ADE decreases from 1.918 to 1.854, 1.752, and 1.588, while FDE decreases from 4.636 to 4.571, 4.383, and 4.089, respectively. Mean jerk and collision rate show a similar overall improvement. Smoothness is not strictly monotonic: LNDDS-2 increases the smoothness cost from 0.047 to 0.053, whereas LNDDS-5, LNDDS-7, and LNDDS-10 reduce it to 0.038, 0.040, and 0.028, respectively. Among the tested settings, LNDDS-10 obtains the best values for all reported traffic metrics, reducing mean jerk from 106.742 to 12.537 and collision rate from 0.407% to 0.255%. Under this HiLo–nuPlan setting, a wider low-noise direct-denoising range is generally associated with lower trajectory error, smoother motion, and fewer vehicle collisions. This conclusion is empirical and should not be generalized beyond the evaluated model, dataset, and sampling configuration without further experiments.

### 4.4. Sensitivity to the Number of Direct-Denoising Steps

**Figure 1:**
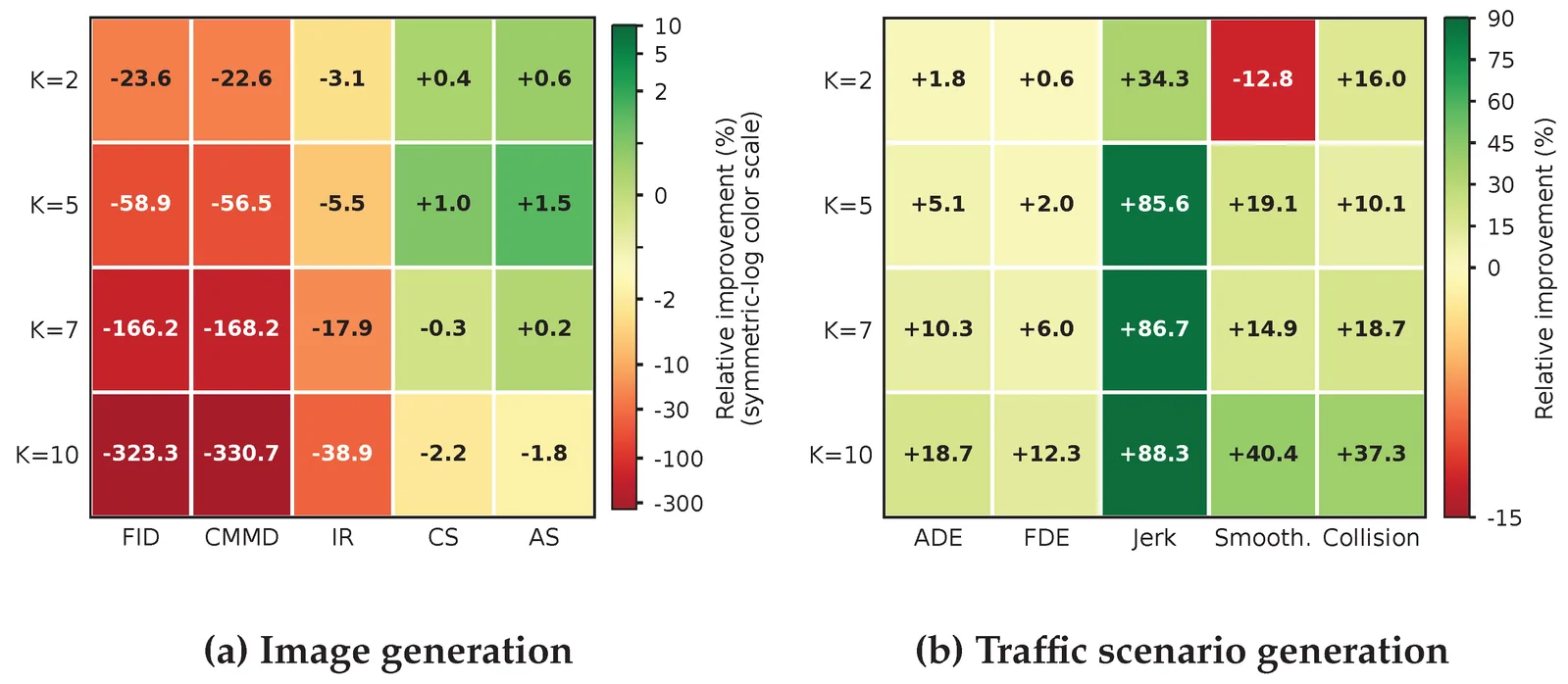
Relative improvement of LNDDS-2, LNDDS-5, LNDDS-7, and LNDDS-10 over the baseline in image generation and traffic scenario generation. Positive values indicate better performance after accounting for the preferred direction of each metric. Image metrics exhibit a non-monotonic and metric-dependent response to *K*, whereas the traffic metrics generally improve as the direct-denoising range increases.

Figure 1 visualizes the relative metric changes reported in Table 2 and Table 3. The figure uses relative improvement instead of raw metric values because the image and traffic metrics have different numerical scales. In image generation, CS and AS initially improve and reach their best values at K=5, whereas FID, CMMD, and IR deteriorate as *K* increases. The degradation becomes substantially larger at K=7 and K=10, indicating that direct denoising is activated before all fine-scale image refinements have stabilized. In traffic generation, ADE, FDE, jerk, and collision rate generally improve as *K* increases, and K=10 produces the best overall results among the evaluated settings. Smoothness shows a small non-monotonic variation at K=2 and K=7, but its best value is also obtained at K=10. These different trends demonstrate that *K* is a domain-dependent hyperparameter rather than a universally transferable constant. In practice, *K* should be selected on a held-out validation set using the primary quality and validity metrics of the target domain.

### 4.5. Low-Noise Mechanism Analysis

Table 4 reports the low-noise mechanism statistics. In the image domain, the averaged $C_{i}$ and $\Delta_{i}$ decrease from the baseline to LNDDS-2 and LNDDS-5, reaching their smallest values of 0.0056 and 0.0088 at K=5. However, they increase to (0.0193,0.0451) at K=7 and (0.0404,0.0992) at K=10. Meanwhile, the final state divergence increases from 0.0111 at K=2 to 0.0279, 0.1733, and 0.3918 as *K* increases to 5, 7, and 10. This rapid increase beyond K=5 is consistent with the degradation of FID, CMMD, and IR in Table 2. In the traffic domain, all four LNDDS settings reduce the DPM++ update deviation relative to the baseline value of 1.3018. The deviation drops to 0.7842 at K=2 and reaches its minimum of 0.0074 at K=5, before increasing moderately to 0.0158 and 0.0286 at K=7 and K=10. The corresponding final state divergence grows with *K*, but this greater deviation remains associated with improved traffic metrics. These results support a limited interpretation: increasing *K* changes both the magnitude and the onset of the sampling-trajectory deviation, while whether this change is beneficial depends on the data domain.

**Figure 2:**
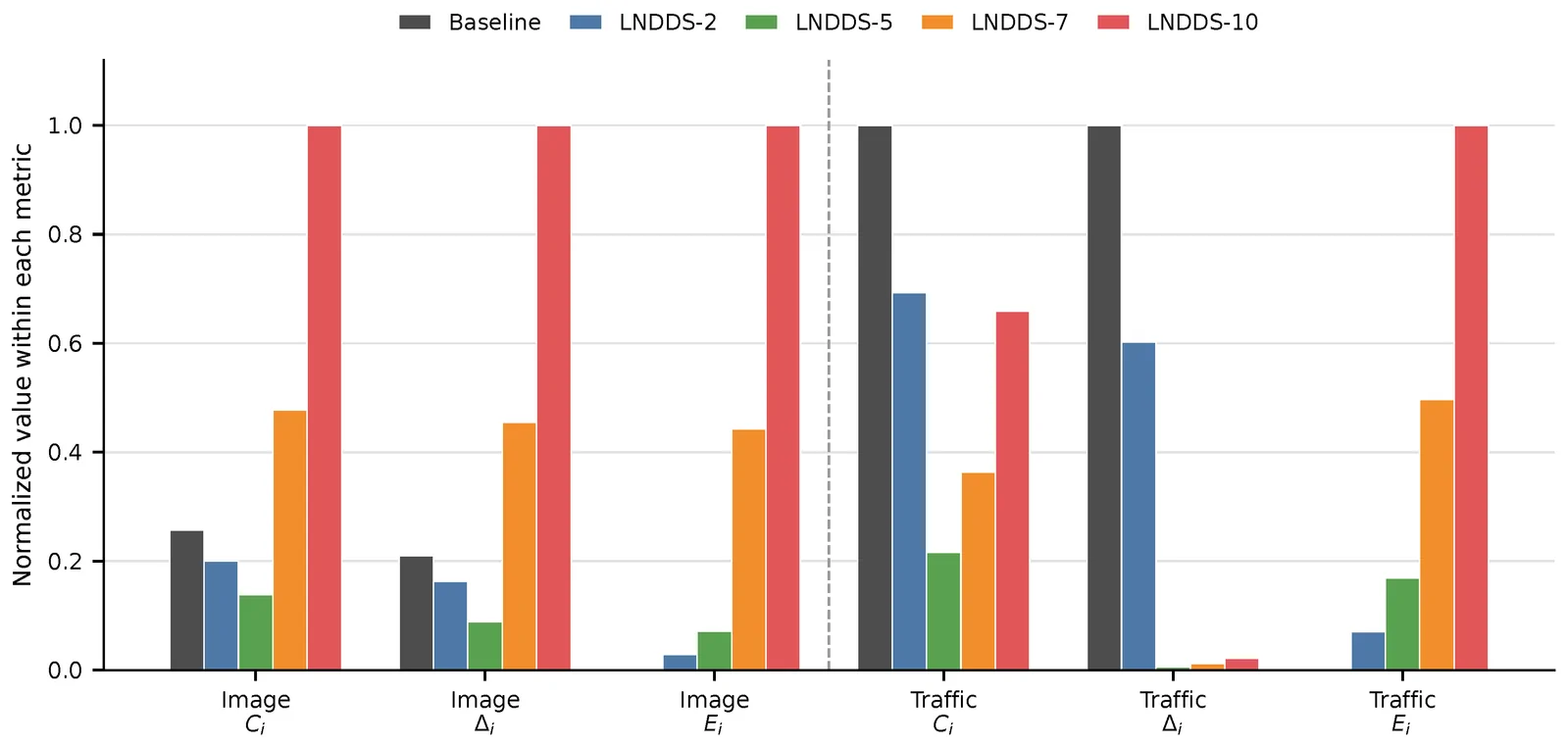
Summary visualization of the low-noise mechanism metrics. The metrics $C_{i}$, $\Delta_{i}$, and final $E_{i}$ describe denoised prediction stability, DPM++ update deviation, and cross-sampler state divergence, respectively. This figure complements Table 4 by showing the relative scale of the low-noise state changes.

Figure 2 provides a visual summary of the mechanism statistics in Table 4. In the image domain, the mechanism metrics initially decrease at K=2 and K=5, but both the update deviation and final state divergence increase sharply at K=7 and K=10. In the traffic domain, all evaluated values of *K* reduce the DPM++ update deviation relative to the baseline, while the final state divergence increases gradually with *K*. These observations are consistent with the domain-dependent trends reported in Table 2 and Table 3.

### 4.6. Robustness to the Number of Sampling Steps

We further evaluate the baseline and LNDDS using 20, 30, and 50 inference steps. This experiment checks whether the observed trend is tied to a single sampling discretization.

Table 5 shows a consistent domain-level difference. In image generation, LNDDS-2 remains closest to the corresponding baseline, while LNDDS-5 provides small improvements in CS and AS at the cost of worse FID, CMMD, and IR. The degradation generally becomes larger at K=7 and K=10, especially when fewer inference steps are used. In traffic generation, all four LNDDS settings reduce ADE, FDE, jerk, and collision rate across 20, 30, and 50 inference steps. Smoothness is not strictly monotonic for every combination of *N* and *K*, but LNDDS-10 provides the strongest overall improvement. The different responses across *K* and *N* further indicate that the effective switching range depends on both the generation domain and the sampling discretization.

### 4.7. Qualitative Comparison

In addition to the quantitative results, we provide qualitative comparisons for both image generation and traffic scenario generation. These examples are used to visually inspect how the low-noise direct update changes the generated outputs. They are not intended to replace the quantitative metrics, but rather to provide complementary evidence for the trends reported in Table 2 and Table 3.

**Figure 3:**
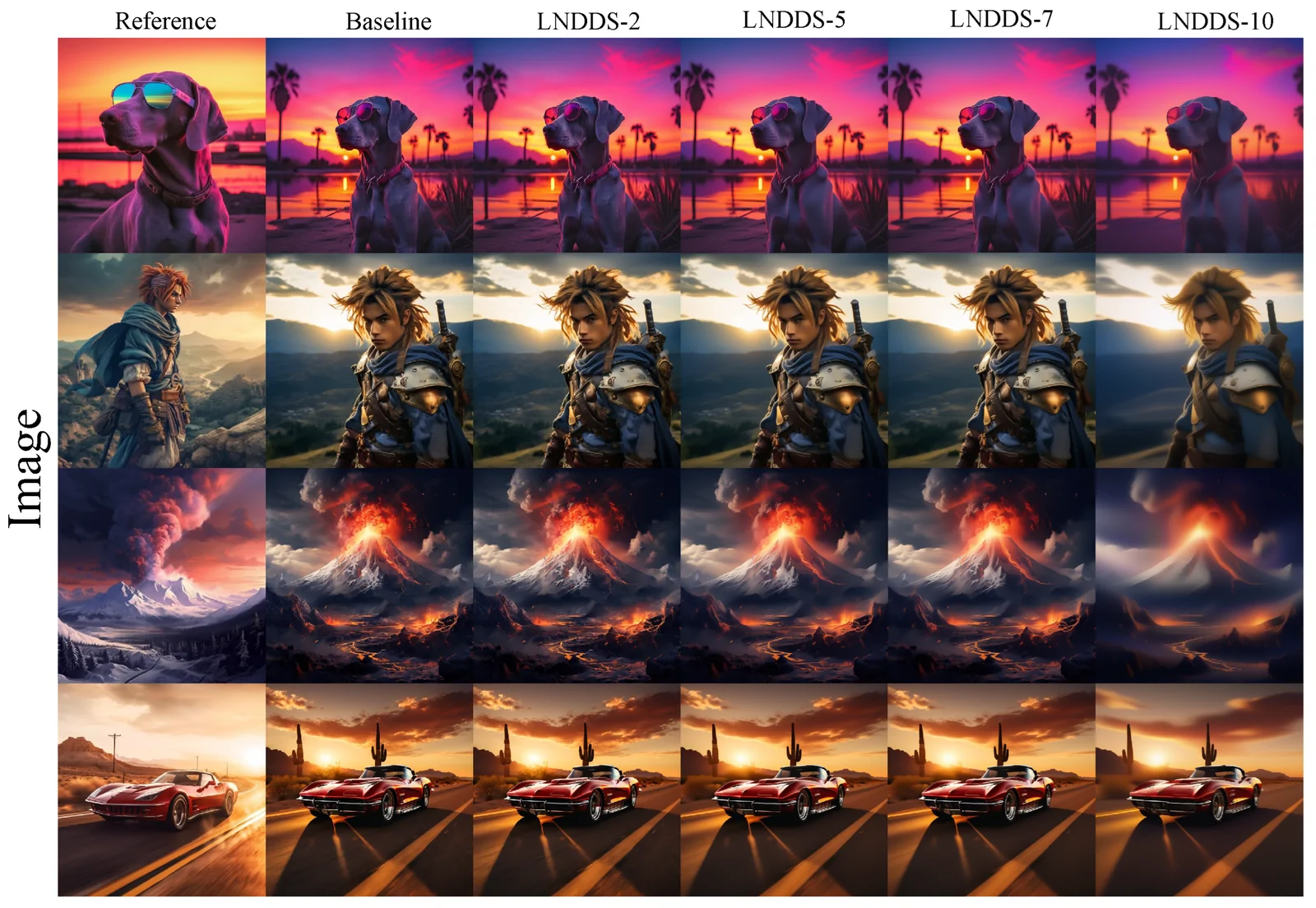
Qualitative comparison of image generation results. Each row uses the same prompt, random seed, initial latent, model weights, and sampling steps. For readability, the columns show the reference image, the baseline sampler, and two representative settings, LNDDS-5 and LNDDS-10; the complete K∈{2,5,7,10} comparison is reported quantitatively. The examples illustrate that LNDDS can change visual appearance in the low-noise stage, but the changes are not uniformly beneficial across image cases.

Figure 3 shows representative image generation examples. The comparison uses the same prompt, seed, and initial latent for all methods, so the visual differences are caused by the low-noise state-update rule. Consistent with Table 2, the qualitative results show that LNDDS may change local visual appearance and overall aesthetics, but this does not necessarily correspond to better distribution-level quality. In particular, using more direct denoising steps can produce larger visual deviations from the baseline, which is consistent with the degradation of FID, CMMD, and IR under LNDDS-10.

**Figure 4:**
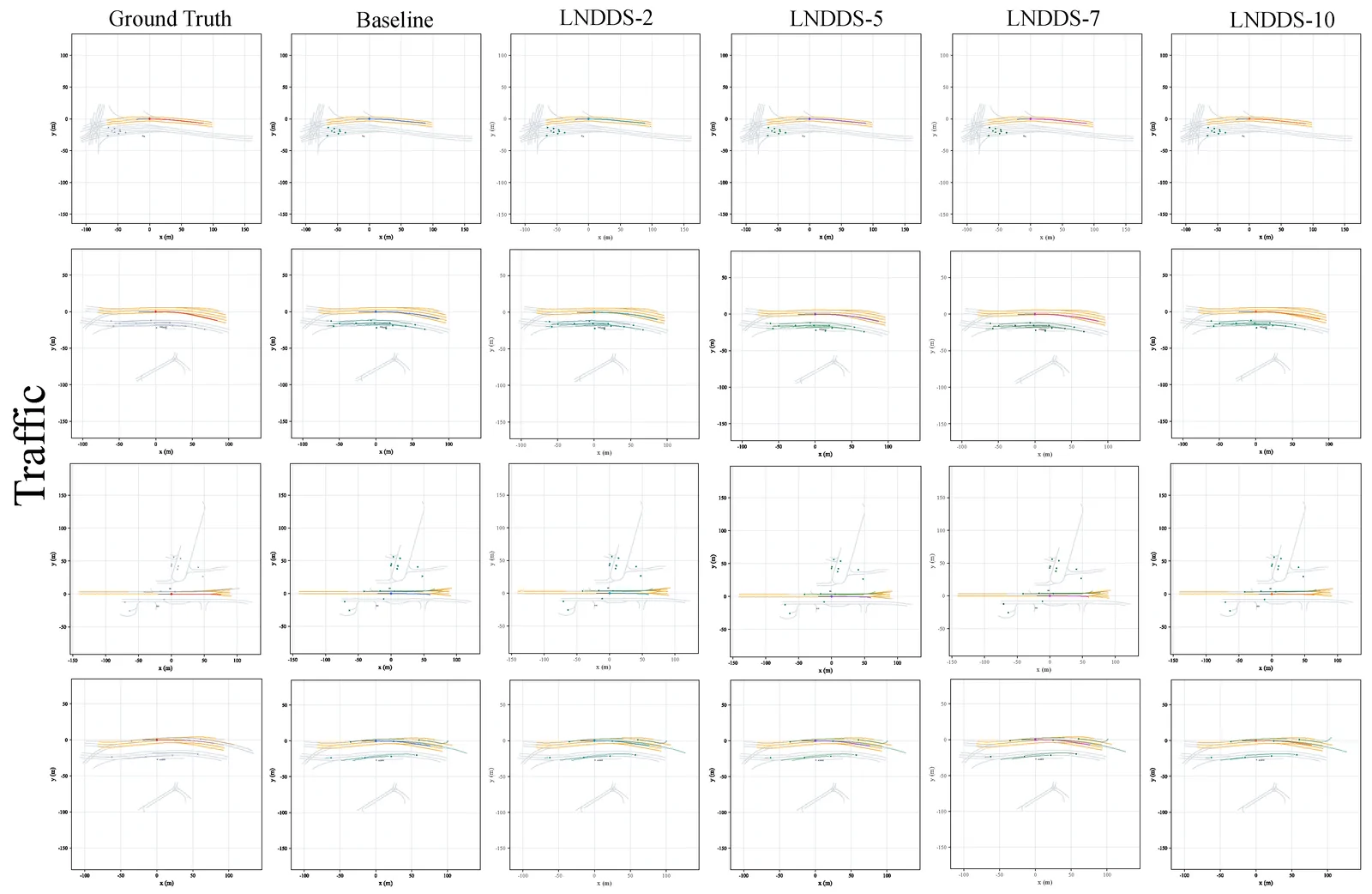
Qualitative Each example uses the same scene condition, map context, agent history, random seed, initial noise, and number of sampling steps. For readability, the columns show the recorded future trajectory, the baseline sampler, and two representative settings, LNDDS-5 and LNDDS-10; all four values of *K* are evaluated quantitatively. The gray and yellow lines represent lane markings, while the other colored lines represent vehicle trajectories generated under different values of *K*. The examples are selected to show typical changes in trajectory smoothness, interaction consistency, and collision tendency.

Figure 4 presents representative traffic trajectory comparisons. Since all methods use the same scene condition and initial noise, the differences mainly reflect the effect of replacing the recursive DPM++ update in the low-noise stage. Compared with the baseline, the LNDDS variants tend to produce smoother trajectories in the shown examples. This visual trend is consistent with the quantitative reductions in jerk, smoothness cost, and collision rate reported in Table 3. However, these qualitative examples should be interpreted together with the aggregate metrics, because individual examples cannot fully characterize the overall behavior distribution.

### 4.8. Discussion

The completed experiments support three restrained conclusions. First, LNDDS does not consistently improve image generation quality on MJHQ-30K. The baseline remains better in FID, CMMD, and IR; LNDDS-2 produces only small changes, and LNDDS-5 obtains the best CS and AS, whereas LNDDS-7 and LNDDS-10 cause increasingly large degradation in most image metrics. Second, in the current HiLo–nuPlan setting, ADE, FDE, jerk, and collision rate generally improve as *K* increases, with LNDDS-10 producing the best overall traffic results among the tested settings. Third, the effect of increasing the number of direct low-noise steps is strongly domain-dependent. A wider switching range is beneficial for the evaluated structured trajectories but detrimental to the evaluated image distribution once *K* exceeds the narrow low-noise region.

Overall, LNDDS should be interpreted as a low-noise state-update strategy whose effect depends on the structure of the generation task. The current results do not support a broad claim that LNDDS is a universal image-quality improvement method. They instead indicate that replacing the recursive DPM++ update in the low-noise stage can be useful for structured trajectory generation, while its benefit for text-to-image generation is limited under the evaluated setting. Consequently, *K* should be selected using a held-out validation set and domain-specific quality metrics rather than treated as a universal constant.

## 5. Conclusions and Limitations

This paper proposes Low-Noise Direct Denoising Sampling (LNDDS) for DPM++ samplers. The method replaces the recursive DPM++ update with the current model-predicted denoised output during the last *K* low-noise steps, while keeping the model architecture, model parameters, noise schedule, total number of sampling steps, and number of function evaluations unchanged. Therefore, the difference between LNDDS and the baseline is caused solely by the state-update rule in the low-noise regime. We evaluate K∈{2,5,7,10} to investigate the sensitivity of the method to the low-noise switching range.

The experimental results demonstrate that the effect of *K* is strongly domain-dependent. On MJHQ-30K image generation, LNDDS-2 slightly improves CLIP Score and Aesthetic Score from 29.348 and 6.817 to 29.472 and 6.861, respectively, but degrades FID, CMMD, and ImageReward. LNDDS-5 achieves the best CLIP Score (29.648) and Aesthetic Score (6.921) among the evaluated settings, while further degrading the distribution- and preference-level metrics. Increasing *K* to 7 and 10 produces progressively larger degradation: FID increases from the baseline value of 7.179 to 19.107 and 30.390, respectively, while ImageReward decreases from 1.111 to 0.912 and 0.679. At K=10, CLIP Score and Aesthetic Score also become worse than the baseline. These results reveal a non-monotonic and metric-dependent response to *K*. A small or moderate direct-denoising range may slightly improve semantic alignment and visual aesthetics, whereas an excessively large *K* can remove low-noise refinement steps that remain important for image generation. Therefore, LNDDS should not be regarded as a general-purpose image-quality improvement method.

In contrast, LNDDS provides clearer benefits in the HiLo–nuPlan traffic scenario generation task. As *K* increases from 2 to 5, 7, and 10, ADE decreases from 1.918 to 1.854, 1.752, and 1.588, respectively, compared with 1.954 for the baseline. FDE similarly decreases from 4.636 to 4.571, 4.383, and 4.089, compared with the baseline value of 4.663. Mean jerk is substantially reduced from 106.742 for the baseline to 70.174, 15.333, 14.220, and 12.537 for LNDDS-2, LNDDS-5, LNDDS-7, and LNDDS-10, respectively. Although smoothness and collision rate do not improve strictly monotonically for every intermediate value of *K*, LNDDS-10 achieves the best overall traffic performance, reducing smoothness cost to 0.028 and collision rate to 0.255%. These results suggest that a wider low-noise direct-denoising range is generally beneficial for the evaluated structured trajectory generation task, where it can reduce trajectory jitter and improve physical consistency.

The mechanism analysis further explains the domain-dependent effect of *K*. In the image domain, the averaged denoised prediction change $C_{i}$ decreases from 0.0104 for the baseline to 0.0081 and 0.0056 for LNDDS-2 and LNDDS-5, respectively. The corresponding DPM++ update deviation $\Delta_{i}$ decreases from 0.0208 to 0.0162 and 0.0088. However, when *K* increases to 7 and 10, $C_{i}$ increases to 0.0193 and 0.0404, while $\Delta_{i}$ increases to 0.0451 and 0.0992. The final state divergence $E_{i}$ also grows from 0.0111 at K=2 to 0.0279, 0.1733, and 0.3918 at K=5, K=7, and K=10, respectively. The sharp increase beyond K=5 is consistent with the degradation of the image-generation metrics.

In the traffic domain, all four LNDDS settings reduce the DPM++ update deviation relative to the baseline value of 1.3018. Specifically, $\Delta_{i}$ decreases to 0.7842, 0.0074, 0.0158, and 0.0286 for K=2, K=5, K=7, and K=10, respectively. Although the final state divergence increases from 0.0053 at K=2 to 0.0127, 0.0374, and 0.0753 as *K* increases, this growing deviation remains associated with improved traffic-generation metrics. These observations show that a larger deviation from the baseline sampling trajectory is not necessarily harmful; its effect depends on whether the removed low-noise recursive updates contribute useful domain-specific refinement. Consequently, LNDDS should be interpreted as a task-dependent low-noise state-update strategy rather than a universally effective sampler improvement.

This work has several limitations. First, the image-generation experiments are limited to Playground v2.5 and MJHQ-30K. Although MJHQ-30K is well aligned with the high-aesthetic-quality text-to-image generation setting considered in this work, evaluation on a single image benchmark is insufficient to establish generality across different image distributions. In particular, the current experiments do not cover commonly used benchmarks such as MS-COCO and ImageNet, nor do they cover additional diffusion models, sampling schedules, and DPM++ variants. Therefore, the reported image-generation results should be interpreted as a controlled evaluation under the Playground v2.5–MJHQ-30K setting rather than evidence of universal superiority over existing solvers.

Second, although K∈{2,5,7,10} provides a broader sensitivity analysis than using only two settings, these discrete values do not exhaustively characterize the optimal switching point. Evaluating every possible value of *K* requires generating and assessing a large number of samples and is computationally expensive. Third, the current switching strategy uses a fixed *K* for all samples and cannot adapt to sample-specific denoised prediction stability, DPM++ update deviation, or domain characteristics. Finally, the traffic experiments are conducted in an open-loop setting, and the effect of the generated scenarios on closed-loop autonomous-driving simulation remains to be evaluated.

Future work will investigate adaptive switching strategies that select *K* or the switching point according to observable low-noise statistics, such as the denoised prediction change $C_{i}$ and the DPM++ update deviation $\Delta_{i}$. We will further evaluate LNDDS on MS-COCO, ImageNet, and other image-generation benchmarks to examine its behavior across different image distributions and task settings. Additional diffusion models, noise schedules, and DPM++ variants will also be considered to determine the scope and limitations of the proposed state-update strategy. Finally, we will extend the traffic evaluation to additional structured-generation datasets and closed-loop autonomous-driving simulation environments.

## Figures and Tables

**Table 1:** Summary of the experimental protocol.

Domain	Model	Dataset	Evaluation Size	Steps	Compared Methods
Image	Playground v2.5	MJHQ-30K	30,000 prompts	30	Baseline, LNDDS-2, LNDDS-5, LNDDS-7, LNDDS-10
Traffic	HiLo	nuPlan	15,000 scenes	20	Baseline, LNDDS-2, LNDDS-5, LNDDS-7, LNDDS-10

**Table 2:** Image generation performance on MJHQ-30K. All methods use the same prompts, random seeds, initial latents, model weights, sampling steps, and number of network evaluations. Best results are shown in bold.

Method	FID ↓	CMMD ↓	IR ↑	CS ↑	AS ↑
Baseline	**7.179**	**0.1001**	**1.111**	29.348	6.817
LNDDS-2	8.872	0.1227	1.077	29.472	6.861
LNDDS-5	11.407	0.1567	1.050	**29.648**	**6.921**
LNDDS-7	19.107	0.2685	0.912	29.258	6.832
LNDDS-10	30.390	0.4311	0.679	28.698	6.691

*Note:* ↓ indicates that lower values are better, while ↑ indicates that higher values are better. Bold values indicate the best performance for each metric.

**Table 3:** Traffic scenario generation results on the held-out nuPlan test set using 20 inference steps. The same HiLo checkpoint, scene conditions, random seeds, initial noise, and sampling steps are used for all methods.

Method	ADE ↓	FDE ↓	Jerk ↓	Smoothness ↓	Collision (%) ↓
Baseline	1.954	4.663	106.742	0.047	0.407
LNDDS-2	1.918	4.636	70.174	0.053	0.342
LNDDS-5	1.854	4.571	15.333	0.038	0.366
LNDDS-7	1.752	4.383	14.220	0.040	0.331
LNDDS-10	**1.588**	**4.089**	**12.537**	**0.028**	**0.255**

*Note:* ↓ indicates that lower values are better, while ↑ indicates that higher values are better. Bold values indicate the best performance for each metric.

**Table 4:** Summary of low-noise mechanism metrics. Values are averaged over the selected low-noise steps for each method.

Domain	Method	$C_{i}$ ↓	$\Delta_{i}$ ↓	Final $E_{i}$
Image	Baseline	0.0104	0.0208	0.0000
	LNDDS-2	0.0081	0.0162	0.0111
	LNDDS-5	0.0056	0.0088	0.0279
	LNDDS-7	0.0193	0.0451	0.1733
	LNDDS-10	0.0404	0.0992	0.3918
Traffic	Baseline	0.0088	1.3018	0.0000
	LNDDS-2	0.0061	0.7842	0.0053
	LNDDS-5	0.0019	0.0074	0.0127
	LNDDS-7	0.0032	0.0158	0.0374
	LNDDS-10	0.0058	0.0286	0.0753

*Note:* ↓ indicates that lower values are better, while ↑ indicates that higher values are better. Bold values indicate the best performance for each metric.

**Table 5:** Robustness to the number of inference steps.

Steps	Method	Image					Traffic				
		IR ↑	FID ↓	CMMD ↓	CS ↑	AS ↑	ADE ↓	FDE ↓	Jerk ↓	Smoothness ↓	Collision (%) ↓
20	Baseline	1.105	17.164	0.1018	29.328	6.814	1.913	4.573	106.833	0.047	0.391
	LNDDS-2	1.095	18.215	0.1182	29.465	6.853	1.880	4.545	70.271	0.033	0.379
	LNDDS-5	1.055	19.777	0.1418	29.658	6.899	1.811	4.478	15.415	0.038	0.342
	LNDDS-7	0.369	43.511	0.4539	27.794	6.243	1.715	4.294	14.322	0.043	0.315
	LNDDS-10	–0.685	79.325	0.9236	24.981	5.248	1.551	3.999	12.666	0.029	0.250
30	Baseline	1.103	16.903	0.1009	29.363	6.805	1.905	4.555	108.990	0.047	0.392
	LNDDS-2	1.104	17.245	0.1078	29.463	6.843	1.887	4.543	75.311	0.052	0.387
	LNDDS-5	1.088	17.748	0.1197	29.600	6.876	1.841	4.504	24.765	0.040	0.355
	LNDDS-7	0.929	26.614	0.2436	29.252	6.801	1.792	4.443	20.082	0.043	0.342
	LNDDS-10	0.676	39.900	0.4287	28.715	6.674	1.710	4.331	13.039	0.033	0.305
50	Baseline	1.106	16.789	0.1013	29.376	6.810	1.902	4.552	109.873	0.046	0.391
	LNDDS-2	1.109	16.892	0.1062	29.436	6.835	1.896	4.551	83.742	0.052	0.385
	LNDDS-5	1.101	17.038	0.1117	29.503	6.848	1.861	4.523	44.533	0.043	0.363
	LNDDS-7	1.092	18.699	0.1294	29.545	6.882	1.843	4.502	32.373	0.051	0.363
	LNDDS-10	1.050	21.167	0.1534	29.657	6.907	1.797	4.454	14.109	0.037	0.338

*Note:* ↓ indicates that lower values are better, while ↑ indicates that higher values are better.

## Data Availability

Data is contained within the article. The original data presented in the study are openly available in MJHQ-30K dataset at https://huggingface.co/datasets/playgroundai/MJHQ-30K/commit/d72535f77a46b6bd05d9d52f2c63d4de115b701f (accessed on 28 July 2026). The implementation code is publicly available at https://gitee.com/liang_zhi_gang/lndds.git (accessed on 28 July 2026).
